# The Development of a Trauma Interventional Radiology Alert Protocol Reduces Time to Vessel Puncture in Cases of Active Hemorrhage

**DOI:** 10.7759/cureus.84173

**Published:** 2025-05-15

**Authors:** Aahad Khan, Akhil Gangisetty, Steve Mitchell, Nick Daughtery, Brent Goslin, William B DeVoe, John Bach

**Affiliations:** 1 Surgery, University of Wisconsin–Madison, Madison, USA; 2 Surgery, OhioHealth Riverside Methodist Hospital, Columbus, USA; 3 General Surgery, OhioHealth Riverside Methodist Hospital, Columbus, USA

**Keywords:** acute care surgery and trauma, acute trauma care, blunt liver trauma, general trauma surgery, interventional radiology-guided embolization, modern trauma, splenic trauma, trauma centers, trauma imaging, trauma patients

## Abstract

Introduction

Trauma continues to be a major cause of death in the United States, with uncontrolled bleeding contributing to a significant portion of trauma-related fatalities. In recent years, the management of hemorrhagic trauma patients has expanded to include interventional radiology (IR). The American College of Surgeons Committee on Trauma recommends that Level 1 and 2 trauma centers ensure IR availability within 60 minutes of the decision to proceed with angiography. Delays in IR intervention are associated with poorer outcomes and increased mortality. To address this, our Level 2 trauma center developed and implemented an institutional protocol involving trauma surgeons, interventional radiologists, residents, trauma advanced practice providers, ED staff, and IR nursing teams to reduce time to intervention for trauma patients with uncontrolled hemorrhage.

Methods

On March 1, 2023, a new institutional protocol was launched to expedite IR intervention in trauma patients with hemorrhage. When such a patient is identified, the trauma team leader (TTL) directly contacts an IR physician to review imaging and determine the need for urgent endovascular therapy. If IR intervention is agreed upon, the TTL places a STAT IR consult, which marks the start time for time tracking. This time period ends when an IR physician achieves vascular access. To accelerate intervention, the TTL informs the primary nurse of the protocol activation, and a trauma vascular IR (VIR) alert is sent via Vocera^®^. The primary nurse and VIR charge nurse coordinate room availability, prepare the patient for transport, and ready the IR suite.

Results

Data were collected prospectively after protocol implementation (beginning March 2023) and retrospectively for the period starting January 2022. The pre-protocol cohort included 11 patients, and the post-protocol cohort included 12 patients. Comparison of the two groups showed a significant reduction in mean consult-to-needle time: 102 minutes ± 39.5 pre-protocol vs. 48.2 minutes ± 12.7 post-protocol (p < 0.001).

Conclusions

Timely VIR intervention is essential for effective hemorrhage control in trauma patients. Transitioning a patient from the trauma bay to the IR suite requires seamless coordination across multiple teams, and delays can negatively impact outcomes. Establishing a standardized institutional protocol can reduce time to intervention by streamlining workflows and minimizing communication-related delays. While our study is limited by a small sample size, ongoing data collection is expected to further support these initial findings.

## Introduction

Trauma is a leading cause of death in the United States, with uncontrolled hemorrhage accounting for 30-40% of trauma-related mortality [1,2]. Prompt control of bleeding is critical and can significantly reduce both mortality and morbidity in trauma patients. Traditionally, surgical intervention was the primary approach to managing hemorrhage. However, with the advancement of minimally invasive techniques, the management of active bleeding has evolved to include interventional radiology (IR), when appropriate [3]. As IR capabilities have expanded, access to angioembolization has become more widespread. Procedures such as hepatic and splenic angioembolization are increasingly used to diagnose and treat arterial injuries, and IR has proven especially valuable for hemorrhage control in anatomically challenging areas, such as the pelvis [4,5].

The American College of Surgeons Committee on Trauma (ACS-COT) recommends that all verified Level 1 and 2 trauma centers be capable of achieving IR vascular access within 60 minutes of the decision to proceed with angiography [6]. Delays in IR intervention are associated with poorer outcomes and increased mortality [7-10]. One of the most commonly cited reasons for delay in angioembolization is the absence of a standardized institutional protocol to guide multidisciplinary coordination [8]. At our institution, we identified several communication breakdowns throughout the angioembolization process, contributing to significant variability in the time from consultation to embolization. In response, we developed an institutional protocol at our Level 2 trauma center involving trauma surgeons, interventional radiologists, surgical residents, trauma advanced practice providers, ED nursing staff, and IR nursing, with the goal of reducing time to intervention for trauma patients with active hemorrhage.

## Materials and methods

**Figure 1:**
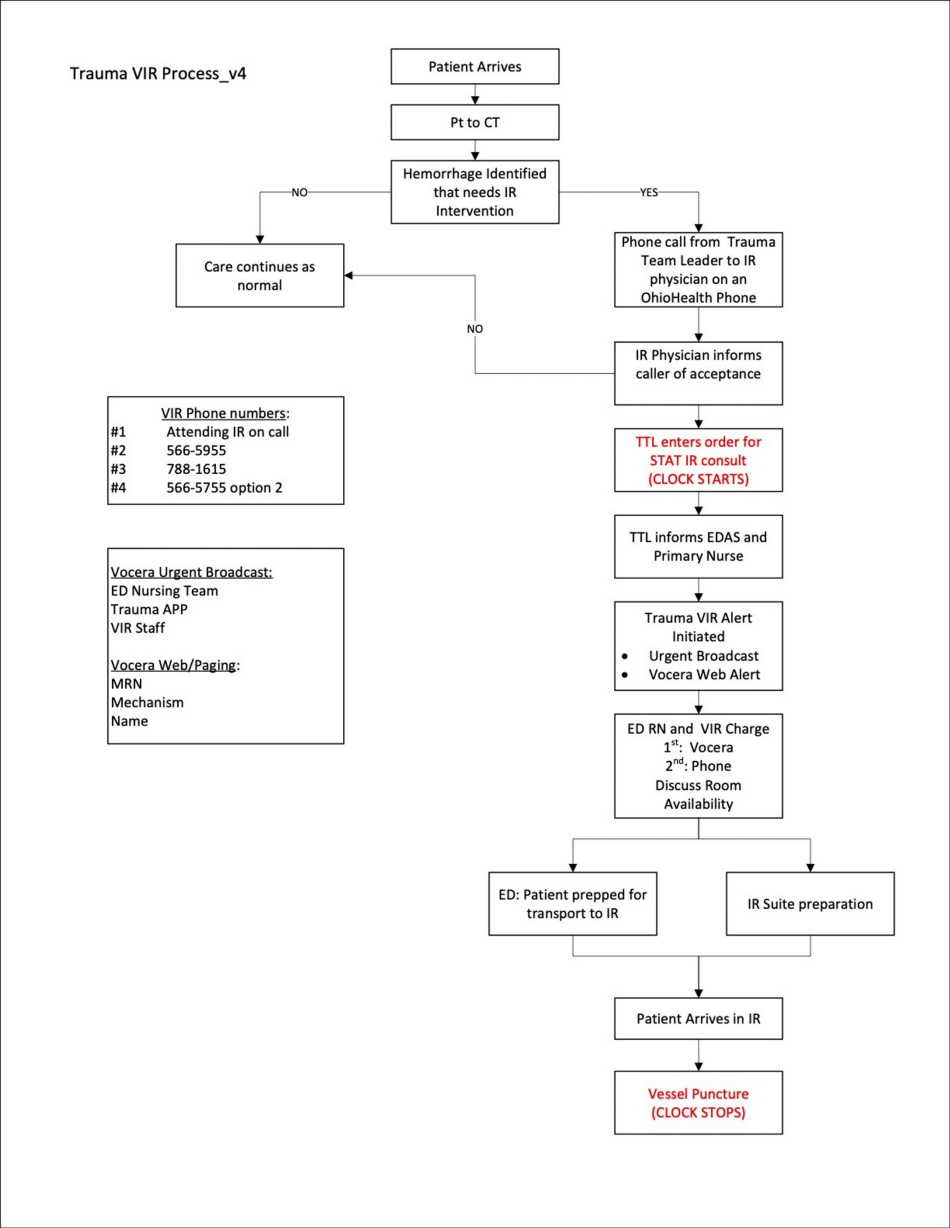
Trauma IR alert protocol APP, advanced practice provider; EDAS, ED assessment; IR, interventional radiology; MRN, medical record number; Pt, patient; RN, registered nurse; TTL, trauma team leader; VIR, vascular interventional radiology Vocera: a hands-free, wearable badge that facilitates communication of notifications and alerts within the hospital; STAT: refers to something that requires immediate or urgent attention

On March 1, 2023, our institution implemented a multidisciplinary protocol designed to reduce the time to IR intervention in trauma patients. The protocol, referred to as the “Trauma IR alert,” was developed through collaboration among trauma surgery, IR, and emergency medicine teams. Prior to its launch, all personnel involved in the care of trauma patients received protocol education to ensure smooth implementation (Figure 1).

The first step of the protocol involves the prompt identification of active hemorrhage in trauma patients that may require IR intervention. For all trauma patients who undergo cross-sectional imaging at our institution, a member of the trauma team is present to identify signs of active hemorrhage. These team members then establish direct communication with an interventional radiologist, who reviews the imaging and decides whether to accept or decline the consult. A “STAT” consult is placed for IR, marking the start time. The time period concludes when the interventional radiologist achieves vascular access (or “vessel puncture”). To reduce time to intervention, the trauma team informs the ED charge nurse and bedside nurse about protocol activation, and a “Trauma vascular IR (VIR) alert” is broadcast via Vocera^®^ to all nursing staff. The bedside nurse and VIR charge nurse then discuss the availability of the angiography suite, and the patient is prepared for transport while the IR suite is simultaneously readied. The patient is subsequently transported by the ER staff and trauma team to the IR department, located directly above the ER.

Trauma patients requiring urgent embolization in the month prior to protocol implementation and one month following protocol implementation were included in the analysis. Eleven patients between January 2022 and February 2023 had severe traumatic injuries that warranted urgent embolization before the standardized protocol was implemented. Twelve trauma patients were identified between March 2023 and June 2023 who were consulted for immediate embolization under the newly implemented protocol. Data were obtained retrospectively for the pre-implementation group and prospectively for the post-implementation group from an in-house trauma database, covering the period from January 2022 to June 2023. Approval from the OhioHealth Office of Human Subjects and the Institutional Review Board was obtained prior to the study’s initiation (IRBNet # 2089945). Two study arms were defined: trauma patients admitted prior to the Trauma IR protocol's implementation and those admitted after its implementation. Metrics collected included demographics, injury severity score, mechanism of injury, injury type, pre-procedure transfusion, anticoagulant/antiplatelet use, injury addressed with angioembolization, time to vessel puncture, length of stay, and mortality. The primary outcome was time to vessel puncture. Statistical analysis was performed using the “jamovi” platform, and chi-squared tests were used for categorical variables, while t-tests were used for continuous variables. A p-value of <0.05 was considered statistically significant.

## Results

The results are summarized in Table 1. The pre-protocol implementation group consisted of 11 patients, while the post-protocol implementation group included 12 patients. The mean ages (65.0 and 59.3 years) and gender distributions were similar, with motor vehicle collisions being the most common mechanism of injury. The most commonly embolized source of bleeding was the splenic vasculature, with 11 cases in total. Comparison of both cohorts revealed a significant difference in the average consult-to-needle time before and after protocol implementation (102 minutes ± 39.5 vs. 48.2 minutes ± 12.7, p < 0.001). There were no statistically significant differences between the groups in sex, age, injury severity score, initial international normalized ratio, or length of stay.

**Table 1:** Results of pre/post-protocol implementation The chi-squared test was used for sex, while the t-test was used for the remaining variables. INR, international normalized ratio; ISS, Injury Severity Score; LOS, length of stay

Variable	Pre-protocol (n = 11)	Post-protocol (n = 12)	p-value
Sex, n (%)			0.827
Female	6 (54.55%)	6 (50.00%)	
Male	5 (45.45%)	6 (50.00%)	
Time to needle (min), mean (SD)	101.82 (39.49)	48.17 (12.70)	<0.001
Age, mean (SD)	65.00 (20.00)	59.25 (25.73)	0.559
ISS, mean (SD)	22.18 (20.05)	23.83 (14.52)	0.822
Initial INR, mean (SD)	1.29 (0.44)	1.18 (0.36)	0.537
LOS (days), mean (SD)	10.82 (7.99)	8.64 (6.02)	0.478

## Discussion

Similar to a previous analysis by Kim et al., our results show that the implementation of a standardized protocol reduced time to intervention, effectively preventing delays caused by variability in communication and the absence of a well-defined pathway for this patient population [10]. Although the study is limited by a relatively small sample size, the average reduction in time to vessel puncture was 54 minutes, which aligns more closely with the ACS-COT’s recommendations [6]. Both the pre- and post-implementation groups seemed to have similar overall injury profiles, as indicated by the ISS. Moreover, the creation of the protocol streamlined the process by setting clear expectations among team members on how to manage hemorrhage in trauma patients who are appropriate candidates for embolization.

The limitations of this study include the small sample size and the retrospective nature of data collection for the pre-protocol implementation group. Additionally, two mortalities were recorded, one from each group. While it is intuitive that more immediate control of active hemorrhage would confer a mortality benefit, we were unable to demonstrate this due to the limited sample size. We also acknowledge the potential for “pre- and post-intervention” bias based on the study design. Other limitations include the fact that this is a single-center study, and the findings may not be generalizable to other institutions with different practices.

Prompt IR intervention plays a crucial role in controlling hemorrhage in trauma patients when angioembolization is the appropriate treatment modality [3]. It is well documented that delays in intervention are generally caused by inadequate or untimely communication between consulting and specialist teams [7-9]. These inefficiencies can ultimately contribute to poor outcomes by exacerbating uncontrolled hemorrhage, leading to worsening shock, increased blood product requirements, and prolonged hospital stays [7,9].

There are several potential future directions based on the results of this study. First, we plan to expand or adapt the current protocol to neighboring trauma centers to assess its generalizability. Secondly, continued data collection is underway with the current protocol to evaluate an increased sample size and investigate the sustained effects beyond six months of protocol implementation. This could allow for further inquiry into secondary clinical outcomes, beyond time to vessel puncture, that are relevant to morbidity and mortality. Finally, ongoing quality measures are in development to refine the process through in-situ simulation and identify potential areas for improvement based on the phase of care.

## Conclusions

The development of an alert protocol for traumatic hemorrhage, deemed best managed by angioembolization, successfully reduced time to vessel puncture in our early experience. Further investigation is needed to determine whether this effect leads to additional clinical benefits, remains sustained, and can be generalized across other trauma centers.

## References

[1] Rhee P, Joseph B, Pandit V, Aziz H, Vercruysse G, Kulvatunyou N, Friese RS (2014). Increasing trauma deaths in the United States. Ann Surg.

[2] Kauvar DS, Lefering R, Wade CE (2006). Impact of hemorrhage on trauma outcome: an overview of epidemiology, clinical presentations, and therapeutic considerations. J Trauma.

[3] Pillai AS, Srinivas S, Kumar G, Pillai AK (2021). Where does interventional radiology fit in with trauma management algorithm?. Semin Intervent Radiol.

[4] Schimmer JA, van der Steeg AF, Zuidema WP (2016). Splenic function after angioembolization for splenic trauma in children and adults: a systematic review. Injury.

[5] Misselbeck TS, Teicher EJ, Cipolle MD, Pasquale MD, Shah KT, Dangleben DA, Badellino MM (2009). Hepatic angioembolization in trauma patients: indications and complications. J Trauma.

[6] American College of Surgeons (2022). Resources for the Optimal Care of the Injured Patient (2022 Standards). https://www.facs.org/media/fdeoqm1h/2022_vrc_injured-patient-standardsmanual_revised_dec-2022.pdf.

[7] Tanizaki S, Maeda S, Matano H, Sera M, Nagai H, Ishida H (2014). Time to pelvic embolization for hemodynamically unstable pelvic fractures may affect the survival for delays up to 60 min. Injury.

[8] Black SR, Sathy AK, Jo C, Wiley MR, Minei JP, Starr AJ (2016). Improved survival after pelvic fracture: 13-year experience at a single trauma center using a multidisciplinary institutional protocol. J Orthop Trauma.

[9] Tesoriero RB, Bruns BR, Narayan M (2017). Angiographic embolization for hemorrhage following pelvic fracture: Is it "time" for a paradigm shift?. J Trauma Acute Care Surg.

[10] Kim C, Niekamp A, Pillai AS (2020). Quality improvement project: improving interventional radiology response times for level I trauma embolization. J Am Coll Radiol.

